# Semi-Analytic Solution and Stability of a Space Truss Using a High-Order Taylor Series Method

**DOI:** 10.3390/ma8052400

**Published:** 2015-05-08

**Authors:** Sudeok Shon, Seungjae Lee, Junhong Ha, Changgeun Cho

**Affiliations:** 1 School of Architectural Engineering, Korea University of Technology and Education, Cheonan 330-708, Korea; E-Mail: sdshon@koreatech.ac.kr; 2 School of Liberal Arts, Korea University of Technology and Education, Cheonan 330-708, Korea; E-Mail: hjh@koreatech.ac.kr; 3 School of Architecture, Chosun University, Gwangju 501-759, Korea; E-Mail: chocg@chosun.ac.kr

**Keywords:** steel space truss, Taylor series method, semi-analytical solution, sinusoidal excitation, beating excitation, attractor, dynamic buckling

## Abstract

This study is to analyse the dynamical instability (or the buckling) of a steel space truss using the accurate solutions obtained by the high-order Taylor series method. One is used to obtain numerical solutions for analysing instability, because it is difficult to find the analytic solution for a geometrical nonlinearity system. However, numerical solutions can yield incorrect analyses in the case of a space truss model with high nonlinearity. So, we use the semi-analytic solutions obtained by the high-order Taylor series to analyse the instability of the nonlinear truss system. Based on the semi-analytic solutions, we investigate the dynamical instability of the truss systems under step, sinusoidal and beating excitations. The analysis results show that the reliable attractors in the phase space can be observed even though various forces are excited. Furthermore, the dynamic buckling levels with periodic sinusoidal and beating excitations are lower, and the responses react sensitively according to the beating and the sinusoidal excitation.

## 1. Introduction

A space truss system has been applied to a variety of structural systems ranging from traditional roof trusses, bridge and reticulated spatial structures to deployable structures. Furthermore, this system has much potential for future use because it is composed of discrete steel members and can form large spaces with a relatively small volume. However, a shallow space truss exhibits unstable phenomena, such as dynamic snapping, due to its nonlinearity.

Studies on structural stability have primarily dealt with static or dynamic buckling of continuous systems, such as shells or arches, in the past, and studies on space trusses have focused on the critical buckling below the static load [1,2,3,4,5,6,7,8,9,10]. Kassimali and Bidhendi [11] investigated the stability problem based on an Eulerian formulation that considered arbitrarily large displacements, and Tada and Suito [12] examined the static and dynamic post-buckling of discrete compressive members using a vibration model. Kim *et al.* [13] considered damping in their study of dynamic buckling of shallow trusses and explained that the structure was more sensitive to indirect snapping than direct snapping [14,15]. Although there are reduction techniques for dynamic analysis [16,17], there are few studies on analytical approaches or the nonlinear dynamic stability of space trusses. Moreover, an exact or an accurate solution of a nonlinear equation must be attained to overcome this problem, and there is a need to analyse the change and characteristics of the periodic orbit. However, it is difficult to obtain the exact solution or an analytical solution of the nonlinear governing equations of a space truss composed of discrete members, and numerical methods [18] as the Newmark-β method and Runge-Kutta method are widely used.

Recently, an analytical approach for both weakly and strongly nonlinear problems has been introduced [19]. The traditional analytic or semi-analytic methods, such as the Taylor’s power series method [20,21], Adomian decomposition method [22,23], the homotopy perturbation method [24,25,26] and so on, have been developed. While these methods are limited in their application and depend on parameters, the Taylor Series Method (TSM) has a long history and is very reliable in terms of offering an analytical solution. Recently, Barrio [21] reported that one of the advantages of the TSM was its easy formulation with the variable-order and variable step-size method, and explained that it was very useful in the analysis of many dynamic systems requiring an accurate analytical solution. Additionally, it has the advantage of allowing the error limit to be calculated so that the computed solution can be assured, and it has also been reported that a very accurate solution can be attained in a very short time by appropriately adjusting for the number of terms and the error limit [20,27,28,29].

Moreover, the accurate solution based on an analytical approach is also needed to deal with the inverse problem or the identification of these steel trusses because the governing equation has a large coefficient and the convergence of the 4th-order Runge-Kutta method (RK4) is O( $h^{4}$). In addition, because TSM, unlike other methods, directly computes the differential coefficients, it is useful when a high-precision solution is required. Furthermore, it is relatively simple in terms of applying the analytical excitation.

Accordingly, the goal of this study is to apply the TSM to a shallow steel space truss and analyse the nonlinear dynamic response. Governing equations are formulated by considering geometric nonlinearity, and a semi-analytical solution is computed by applying the high-order TSM. This paper is organised as follows. Section 2 discusses the formulation of the nonlinear governing equation and the theoretical analysis technique of TSM. Section 3 obtains a semi-analytical solution of the governing equation of a single-free-node (SFN) model and investigates the dynamic instability of SFN model under the various loads, *i.e.*, step, sinusoidal and beating load. The effect of changing damping coefficients is also considered. Section 4 investigates the unstable behaviour of a double-free-node (DFN) model in consideration with an initial imperfection. Lastly, Section 5 proposes conclusions of this study.

## 2. A Discrete Nonlinear Dynamic System and the Taylor Series Method (TSM)

### 2.1. Formulation of Nonlinear Motion Equations of a Space Truss

The dimension for the 3D bar element is defined using a local system (x,y,z) and a global system (X,Y,Z), and the nodal vectors are assumed to be f,d,F, and D with 3 degrees of freedom (DOF) per node. Here, f and d as well as F and D denote the force and displacement vectors of the local and global system, respectively. Each vector in the local and global systems can be transformed by matrix T. The displacement function represented by the nodal vector of the element is defined with a Lagrangian interpolation function, $N_{i}$ and $N_{j}$, as:

(1) $$u=\left[N_{i}I_{3}\;\vdots \;N_{j}I_{3}\right]T^{T}D$$

Using large deformation theory, the strain–displacement relationship of an elastic material is assumed to be as follows [8]:

(2) $$\epsilon =\frac{du}{dx}+\frac{1}{2}\left\{{\left(\frac{du}{dx}\right)}^{2}+{\left(\frac{dv}{dx}\right)}^{2}+{\left(\frac{dw}{dx}\right)}^{2}\right\}$$

The following applies the principle of virtual work to obtain the stiffness equations of the element:

(3) $$\text{δ}d^{T}f=\int^{\text{​}}\text{δ}\epsilon^{T}\sigma dV$$

When the nodal vector in the global system is substituted and integrated, the following equation is the result:

(4) $$\text{δ}D^{T}F=A\;E\;l\;\left(\text{δ}\epsilon^{T}\epsilon \right)$$

where A,E and l are an area, elastic modular and a length of each member, respectively. δϵ and ϵ are expressed in the following equations by obtaining the differential of the displacement functions and substituting it into the above relational equations:

(5) $$\epsilon =UT^{T}D+\frac{1}{2}D^{T}K_{s}D$$

(6) $$\text{δ}\epsilon =UT^{T}\text{δ}D+D^{T}K_{s}\text{δ}D$$

The matrices,U and $K_{s}$, in the above equations are defined as:

(7) $$U=\left\{N_{i,x}\;0\;0\;\vdots \;N_{j,x}\;0\;0\;\right\}$$

(8) $$K_{s}=\frac{1}{l^{2}}\left[\begin{array}{cc} I_{3} & -I_{3}\\ -I_{3} & I_{3} \end{array}\right]$$

The following stiffness equation is obtained by substituting each term of Equation (4) and solving for the nodal displacement vector using the global system:

(9) $$F=EAl\left\{TU^{T}UT^{T}D+\left(K_{s}DUT^{T}+\frac{1}{2}TU^{T}D^{T}K_{s}\right)D+\frac{1}{2}K_{s}DD^{T}K_{s}D\right\}$$

Examining the terms on the right hand side of the above equation, the first term is the first-order, the second and third terms are the second-order, and the last term is the third-order term of the unknown displacement vector. Accordingly, the discrete nonlinear dynamic system is simplified and expressed in the equation below and includes the mass and damping from the above Equation (9).

(10) $$M\ddot{D}+C\dot{D}+\left\{K_{1}+K_{2}(D)+K_{3}(D^{2})\right\}D=F$$

where M is the lumped mass matrix, C is the damping matrix, and $K_{1},K_{2}(D),K_{3}(D^{2})$ are the stiffness matrices for each degree of the terms in Equation (9).

### 2.2. High-Order Taylor Series Method (TSM)

Equation (10) can be expressed in the equation bellow and defined as the following to attain the solution for an initial-value problem of the governing equations using the TSM and an approximate power series solution:

(11) $$\ddot{D}=f\left(t,\dot{D},D\right),\;\;t\in I,\;\;\;D=D(t)\in R^{n}$$

(12) $$D\left(t_{0}\right)=D_{0},\;\dot{D}\left(t_{0}\right)=D_{1}$$

where I is an open interval containing $t_{0},f$ is a sufficiently smooth function on $I\times R^{n}\times R^{n}$. D(t) is an analytic function, which can be expressed by:

(13) $$D\left(t\right)=\overset{\infty }{\underset{n=0}{\sum }}\frac{D^{\left(n\right)}\left(t_{0}\right)}{n!}{\left(t-t_{0}\right)}^{n}$$

(14) $$D\left(t\right)=\overset{n}{\underset{k=0}{\sum }}\frac{D^{\left(k\right)}\left(t_{0}\right)}{k!}{\left(t-t_{0}\right)}^{k}+R\left(n,t,t_{0}\right)$$

When an analytical series solution is to be obtained for the *n*th-degree term, the remainder term, $R\left(n,t,t_{0}\right)$, is expressed as the following, and $c^{*}$, which satisfies the equation below, exists in the open interval. Additionally, the error is defined by Equation (16).

(15) $$R\left(n,t,t_{0}\right)=\frac{D^{\left(n+1\right)}\left(c^{*}\right)}{\left(n+1\right)!}{\left(t-t_{0}\right)}^{n+1}$$

(16) $$\left|R\left(n,t,t_{0}\right)\right|\leq \frac{1}{\left(n+1\right)!}max_{t_{0}\leq t\leq t_{0}+t_{h}}\left|D^{\left(n+1\right)}\left(t\right)\right|\;{t_{h}}^{n+1},\;\;\;\;\;t_{0}\leq t\leq t_{0}+t_{h}$$

TSM defines the solution of Equation (10) as an *n*th-degree series except for the R-term in Equation (14) and obtains the solution of coefficients of each differential, $D^{\left(k\right)}(t_{0})$, from Equation (10). The computed solution has the precision of the solution within the error range of Equation (16) in the defined range of [$t_{0},t_{0}+t_{h}$]. Here, it is efficient to use order n and step-size $t_{h}$ in Equation (16) as the parameters for the multi-step solution, and the variable order (VO) and variable step-size (VS) scheme adjusts for n and $t_{h}$, respectively. Also, the two parameters, n and $t_{h}$, can determine the error limit, and the accuracy of and the time to compute the solution are determined by these two parameters. This study defines the solution of Equation (10) as a finite Taylor series as expressed in Equation (17) and computes the solution in multiple steps.

(17) $$D_{i}\left(t\right)=\overset{n}{\underset{k=0}{\sum }}\frac{D_{i}^{\left(k\right)}\left(t_{0i}\right)}{k!}{\left(t-t_{0i}\right)}^{k}\;,\;\;\;\;\left(\;t_{0i}\leq t\leq t_{0i}+t_{h}\;\right)\;\;\;\;\;i=1,2,\ldots ,total\;step$$

where the initial value pertaining to the *i*th-step can be found by the previous step.

## 3. A Single-Free-Node (SFN) Steel Space Truss Model

**Figure 1:**
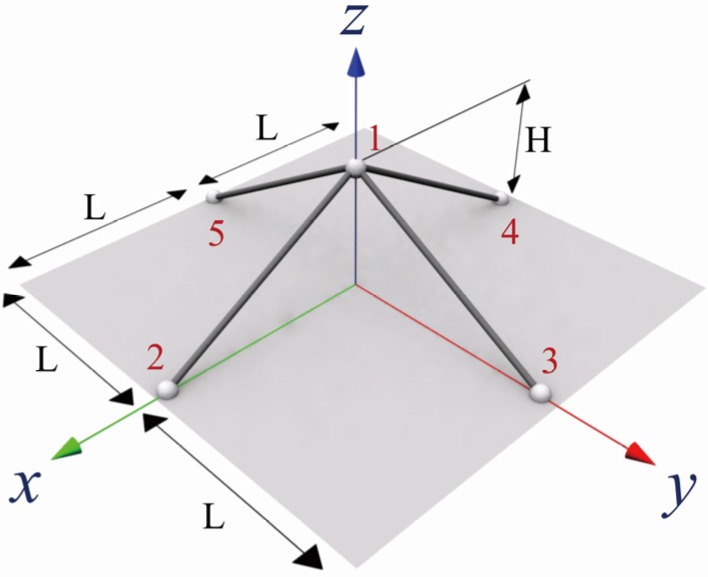
Shape of single-free-node (SFN) model.

This chapter discusses the dynamic analysis and instability of SFN model under step, sinusoidal and beating excitations. SFN model, shown in Figure 1, is composed of five nodes and four elements, and only the top node is free with the others being clamped. This model is widely used to investigate dynamic snapping. In the case of the model, the governing equation, described by Equation (18), can be easily induced by Equation (10), and the coefficients of the differentials $D^{\left(k\right)}(t_{0})$ can be obtained from Equation (18). For example, $D^{\left(k\right)}(t_{0})$ is described by Equation (18a~e) for order n=5. In this equation, the stiffness terms are $K_{1}=4{\text{EAH}}^{2}/L^{3}$, $K_{2}=12\text{EAH}/2L^{3}$ and $K_{3}=4\text{EA}/2L^{3}$. Where, K and A are Young’s modulus and the cross-sectional area of the elements, respectively. H and L are the height and the half-width of SFN model as shown in Figure 1.

(18) $$m\ddot{D}+c\dot{D}+K_{1}D+K_{2}D^{2}+K_{3}D^{3}=F_{0}$$

(18a) $$D^{\left(0\right)}\left(t_{0}\right)=D_{0}$$

(18b) $$D^{\left(1\right)}\left(t_{0}\right)={\dot{D}}_{0}$$

(18c) $$D^{\left(2\right)}\left(t_{0}\right)=-2hw_{0}{\dot{D}}_{0}-w_{0}^{2}D_{0}-\frac{K_{2}}{m}D_{0}^{2}-\frac{K_{3}}{m}D_{0}^{3}+\frac{F_{0}}{m}$$

(18d) $$D^{\left(3\right)}\left(t_{0}\right)={\left(2hw_{0}\right)}^{2}{\dot{D}}_{0}+2hw_{0}^{3}D_{0}+2hw_{0}\frac{K_{2}}{m}D_{0}^{2}+2hw_{0}\frac{K_{3}}{m}D_{0}^{3}-2hw_{0}\frac{F_{0}}{m}-\frac{K_{2}}{m}D_{0}{\dot{D}}_{0}-w_{0}^{2}{\dot{D}}_{0}-3\frac{K_{3}}{m}D_{0}^{2}{\dot{D}}_{0}$$

(18e) $$D^{\left(4\right)}\left(t_{0}\right)=-{\left(2hw_{0}^{2}\right)}^{2}D_{0}-w_{0}^{2}\frac{F_{0}}{m}-6\frac{K_{3}}{m}D_{0}{\dot{D}}_{0}^{2}-2\frac{K_{2}}{m}{\dot{D}}_{0}^{2}+w_{0}^{4}D_{0}-{\left(2hw_{0}\right)}^{3}{\dot{D}}_{0}+{\left(2hw_{0}\right)}^{2}\frac{F_{0}}{m}+3{\left(\frac{K_{3}}{m}\right)}^{2}D_{0}^{5}+2{\left(\frac{K_{2}}{m}\right)}^{2}D_{0}^{3}+4hw_{0}^{3}{\dot{D}}_{0}+3w_{0}^{2}\frac{K_{2}}{m}D_{0}^{2}+4\;w_{0}^{2}\frac{K_{3}}{m}D_{0}^{3}-{\left(2hw_{0}\right)}^{2}\frac{K_{2}}{m}D_{0}^{2}-{\left(2hw_{0}\right)}^{2}\frac{K_{3}}{m}D_{0}^{3}+5\frac{K_{2}}{m}\frac{K_{3}}{m}D_{0}^{4}-3\frac{K_{3}}{m}\frac{F_{0}}{m}D_{0}^{2}-2\frac{K_{2}}{m}\frac{F_{0}}{m}D_{0}+8hw_{0}\frac{K_{2}}{m}D_{0}{\dot{D}}_{0}+12hw_{0}\frac{K_{3}}{m}D_{0}^{2}{\dot{D}}_{0}$$

where $w_{0}$ is the natural angular frequency, $w_{0}=\sqrt{K_{1}/m}$ , with the damping coefficient $h=c/2mw_{0}$, and initial values $D\left(t_{0}\right)=D_{0}$ and $\dot{D}\left(t_{0}\right)={\dot{D}}_{0}$. Accordingly, if the Taylor series is expanded at each step using the above coefficients, an approximate analytical solution can be attained, and the number of terms increases as the order increases. This study used a relatively high order n and a smaller step-size $t_{h}$. In this paper, the adopted model has the following structural information: the material density (ρ), Young’s modulus (E) and the cross-sectional area (A) are $7.85\times 10^{\text{−3}}{\text{kg/m}}^{3}$, $2.06\times 10^{\text{5}}\text{MPa}$ and $1.12{\text{cm}}^{2}$, respectively. The shape parameter μ (=H/2L) is defined by the rise (H) to span (2L=10 m) ratio.

### 3.1. Dynamic Response under a Step Excitation

The analytical solution of the model directly using TSM can be obtained by calculating the coefficients of the differentials, similar to Equation (18). In this case, the order *n* is 7, the rise-span ratio is μ=0.1 and damping is not considered. Also, the step excitation $F_{0}$ is applied.

**Figure 2:**
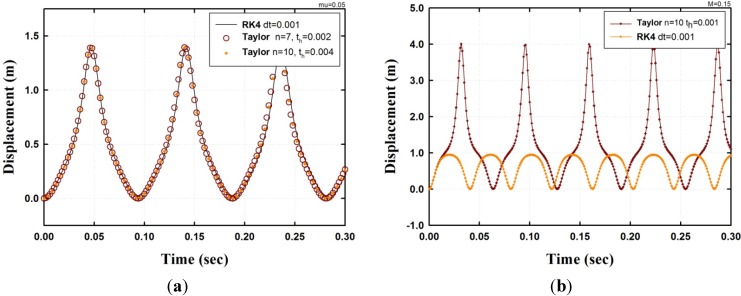
Comparing the result from SFN model using TSM with the result from RK4; (**a**) Displacement μ=0.05, $F_{0}=\text{200 kN}$; (**b**) Displacement μ=0.15, $F_{0}=\text{3000 kN}$.

As shown in Figure 2, the two cases that use high-order TSM are compared with the result of the 4th order Runge-Kutta method (RK4). One case is the model when μ=0.05 at $F_{0}=\text{200 kN}$ (see Figure 2a), and the other model is when μ=0.15 at $F_{0}=\text{3000 kN}$ (see Figure 2b).

In the first case, shown in Figure 2a, the time-step is set to 0.0005 for the RK4, and the order *n* and step size $t_{h}$ of the Taylor method are 7 and 10 and 0.002 and 0.004, respectively. As shown in the figure, the results of the Taylor method agree with the result from the RK4. As mentioned earlier, the increasing order can reduce the errors; however, reducing the step-size can also result in accurate results, which means that an accurate solution with a relatively long time-step can be obtained with a high order.

In the second case, shown in Figure 2b, the result shows that to obtain an accurate solution, the area near the dynamic buckling load is more sensitive and difficult than the other state. In this case, the time parameter of both high-order TSM and the RK4 was set at the same time-step, $t_{h}=0.001$, and the order is n=10. As shown in Figure 2b, the result of the high-order TSM is different from the result of the RK4 and corresponds to the state of post-buckling, whereas the result of the RK4 is pre-buckling. However, increasing $F_{0}$ by only a small amount, the dynamic snap-buckling will also appear in the case of the RK4. Therefore, to obtain a more accurate solution, using the analytical approach with a high order of the differential equations as in TSM would be appropriate.

To investigate the dynamic instability, let us consider another case with μ=0.1 and h=0.0. The parameters for TSM are set at and n=10 and $t_{h}=0.0005$.

**Figure 3:**
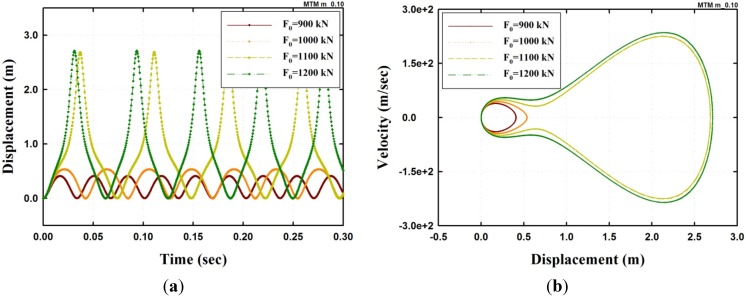
Dynamic analysis results of an undamped SFN model under a step excitation (μ=0.1, h=0.0): (**a**) Displacement; (**b**) Trajectory of the phase space.

First, the time history of the model with $F_{0}=\text{900,1000,1100 and 1200 kN}$ is shown in Figure 3. Figure 3a shows that the period of the model gradually increases up to when the load $F_{0}=\text{1000 kN}$ and then decreases afterwards. The maximum displacement is suddenly amplified at $F_{0}=\text{1100 kN}$ and does not vary in proportion to $F_{0}$ because of the effect of the geometrical nonlinearity. In addition, the rapidly changing maximum displacement as $F_{0}$ increases, between 1000 and 1100 kN, is expected as the critical point, and the $F_{0}$ at the point refers to the dynamic buckling load under a step excitation [1,14]. Figure 3b shows the trajectory in the phase space to observe the attractor before and after dynamic buckling. In the phase space of the figure, the shape of the trajectory is changing from a limit cycle attractor with a single centre point to a limit cycle attractor with two centres.

**Figure 4:**
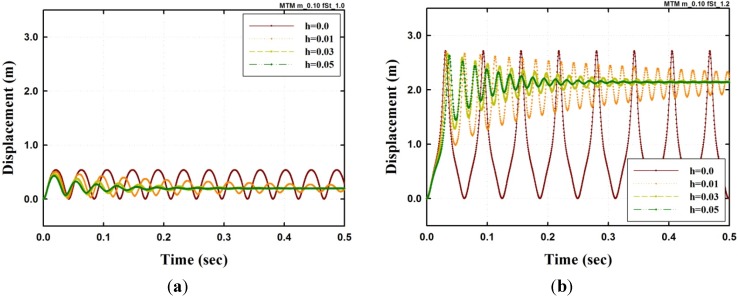
Dynamic analysis results of a damped SFN model under a step excitation (μ=0.1): (**a**) Displacement $F_{0}=\text{1000 kN}$; (**b**) Displacement $F_{0}=\text{1200 kN}$.

**Figure 5:**
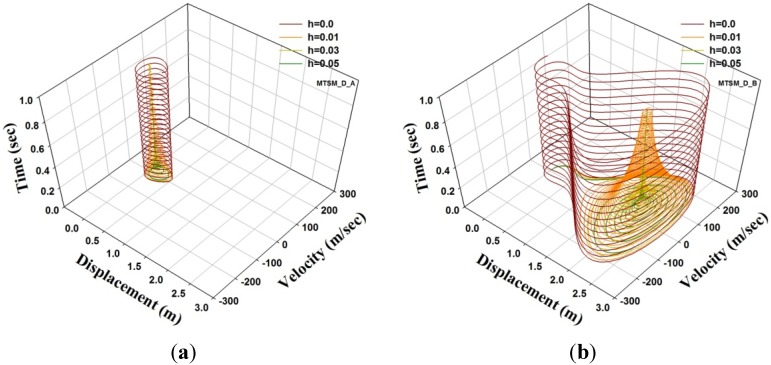
Phase space of a damped SFN model under a step excitation (μ=0.1): (**a**) Extended phase diagram, $F_{0}=\text{1000 kN}$; (**b**) Extended phase diagram, $F_{0}=\text{1200 kN}$.

Next, the results of the model with different damping coefficients *h* are shown in Figure 4 with h=0.01, 0.03 and 0.05. Here, Figure 4a,b shows the result of a pre-buckling load with $F_{0}\;=\;1000\;\text{kN}$ and a post-buckling load with $F_{0}\;=\;1200\;\text{kN}$, respectively. The figures indicate that the curves converge well even with post-buckling. The trajectory in the phase space is shown in Figure 5. In the case $F_{0}=\text{1000 kN}$, the trajectory converged to near the centre point at the trajectory, as shown in Figure 5a. The trajectory when $F_{0}=\text{1200 kN}$ converges on the other centre in the limit cycle attractor, as shown in Figure 5b. However, in this case, it is possible to have two points of the system converge to a limit set, and these fixed point attractors and trajectories in the phase space are sensitive to the initial condition [14].

### 3.2. Dynamic Response under a Periodic Excitation

**Figure 6:**
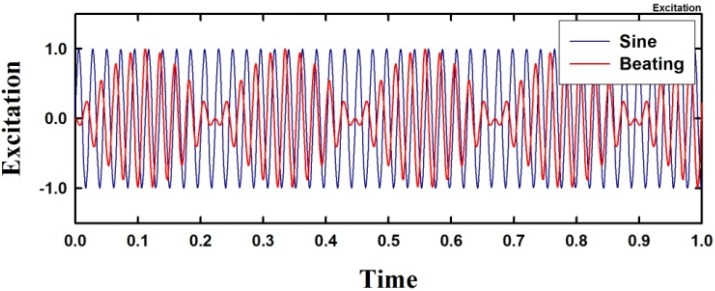
Sinusoidal and beating excitations excitation (α=1.0, β=1.0).

Periodic excitation, such as a sinusoidal and a beating excitation, is defined in Equations (19) and (20), and is shown in Figure 6. In the equations, $w_{0}$ is the natural angular frequency of the analysis model and the periodic parameters α and β are introduced and are applied in the model to investigate the dynamic response under periodic excitations. Let us consider the model with a rise-span ratio of μ=0.1 and a damping coefficient of h=0.0.

(19) $$F=F_{0}\cdot sin\left(\alpha w_{0}t\right)$$

(20) $$F=F_{0}\cdot 0.5\left\{cos\left(\text{α}w_{0}t\right)-cos(\text{α}(1-\text{β})w_{0}t)\right\}$$

**Figure 7:**
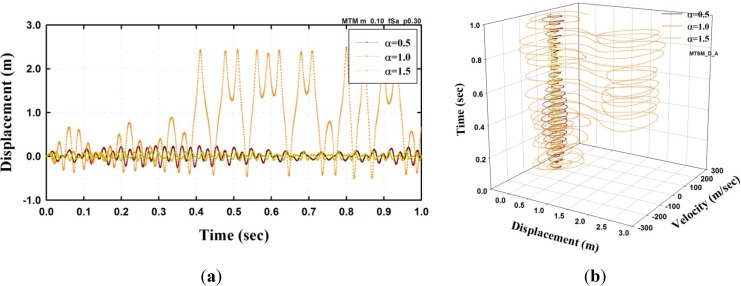
Dynamic analysis results of SFN model under a sinusoidal excitation (μ=1.0, α=1.0, $F_{0}=\text{402 kN}$): (**a**) Displacement (**b**) Trajectory in the phase space.

Figure 7 shows the result of the sinusoidal excitation with $F_{0}=\text{402 kN}$. The analysis result indicated that when α=1.0, the amplitude of the displacement was the greatest, as shown in the Figure 7a, and dynamic buckling was observed. However, dynamic buckling did not occur when α=0.5 and 1.5. These results are depicted in the phase space of Figure 7b. The strange attractor for α=1.0 was formed, as shown in the figure, and the amplitude of response conspicuously increased. In particular, α=1.0 indicates that the frequency of excitation is the same as the first natural angular frequency of the adopted model.

**Figure 8:**
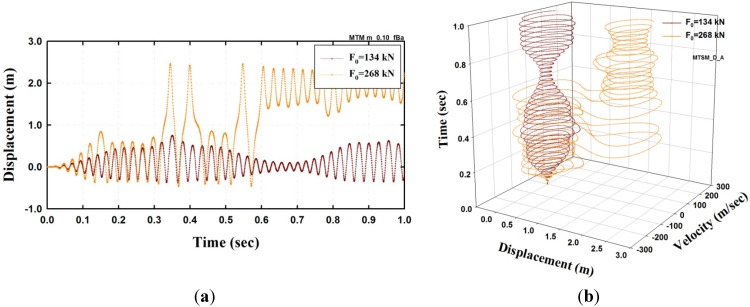
Dynamic analysis results of SFN model under a beating excitation (μ=0.1, α=0.1, β=0.1). (**a**) Displacement; (**b**) Trajectory in the phase space.

In the case of the model under a beating excitation, the periodic parameters, α=1.0 and β=0.1, are considered. Here, α and β represent two periods of the beating excitation. Figure 8 shows the analysis results when $F_{0}=\text{134 and 268 kN}$ = . The amplitude of the displacement markedly increases when $F_{0}=\text{268 kN}$, as shown in Figure 8a. This result can be easily observed from the trajectory of the phase space of Figure 8b, and the change of trajectory is manifested as a strange attractor when $F_{0}=\text{268 kN}$.

### 3.3. Dynamic Instability and Buckling Load

Dynamic instability with different rise-span ratios μ of a shallow SFN model under a step excitation is investigated. Five ratios of μ were used: 0.05, 0.1, 0.15, 0.2 and 0.25. Here, the static critical buckling load level $sP_{\mathrm{cr}}$ is shown in Table 1, and the dynamic buckling load was determined using the Budiansky-Roth criterion [1,14].

**Table 1:** Static buckling load of SFN model ($sP_{\mathrm{cr}}$).

***Μ***	0.05	0.1	0.15	0.2	0.25
**sP_cr_ (kN)**	175	1340	4214	9098	15886

In the case of the undamped model, the dynamic buckling load $dP_{\mathrm{cr}}$ is shown in Table 2. The result shows that the $dP_{\mathrm{cr}}$ increases as μ increases, and the non-dimensional load $dP_{\mathrm{cr}}/sP_{\mathrm{cr}}4$, is approximately 77% of the static buckling. Next, the dynamic buckling load of the damped model, h=0.5, is shown in Table 3. The table shows that $dP_{\mathrm{cr}}$ varies in proportion to the increase in the damping coefficient (*h*), and the dynamic buckling was approximately 83% of the static buckling.

**Table 2:** Dynamic buckling load of SFN model ($dP_{\mathrm{cr}}$) (h=0.0).

***Μ***	0.05	0.1	0.15	0.2	0.25
**dP_cr_ (kN)**	136	1032	3244	7004	12229
**dP_cr_**/**sP_cr_**	0.7771	0.7703	0.7698	0.7698	0.7698

**Table 3:** Dynamic buckling load of SFN model ($dP_{\mathrm{cr}}$) (h=0.5).

***Μ***	0.05	0.1	0.15	0.2	0.25
**dP_cr_ (kN)**	146	1106	3479	7511	13113
**dP_cr_**/**sP_cr_**	0.8343	0.8256	0.8256	0.8255	0.8255

**Figure 9:**
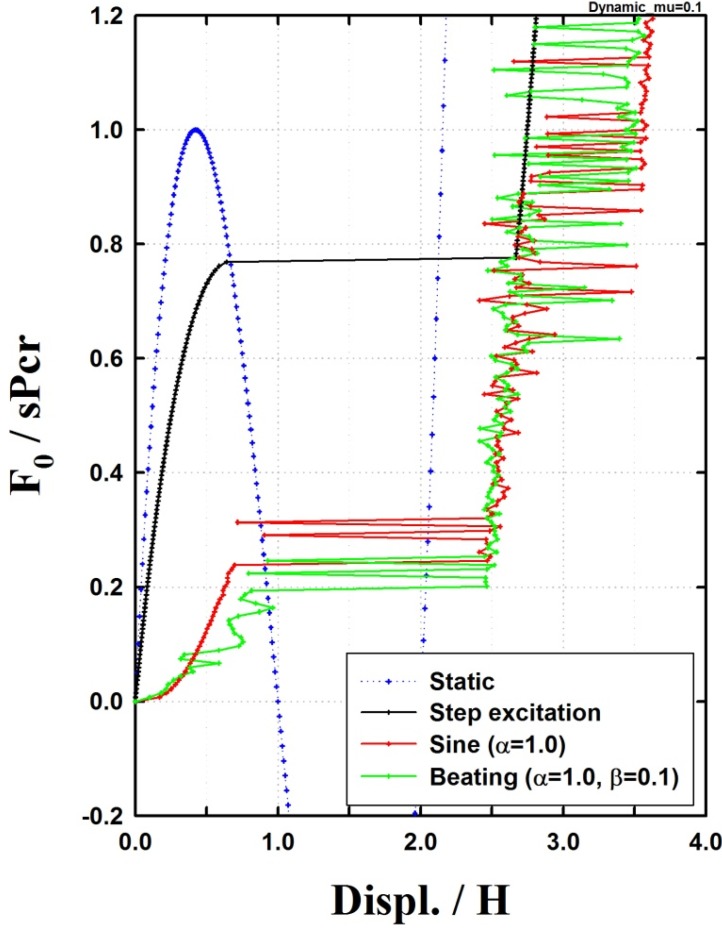
Maximum displacement response under various excitations (μ=0.1).

Figure 9 compares the dynamic buckling load of the beating excitation with that of the static buckling and step and sinusoidal excitations. The figures show that the beating excitation and the sinusoidal excitation resulted in a sensitive change in the maximum displacement. Additionally, the lowest buckling load was observed with the beating excitation followed by the sinusoidal excitation, step excitation, and static buckling in an increasing order of buckling load.

## 4. Double-Free-Nodes (DFN) Steel Space Truss Model

**Figure 10:**
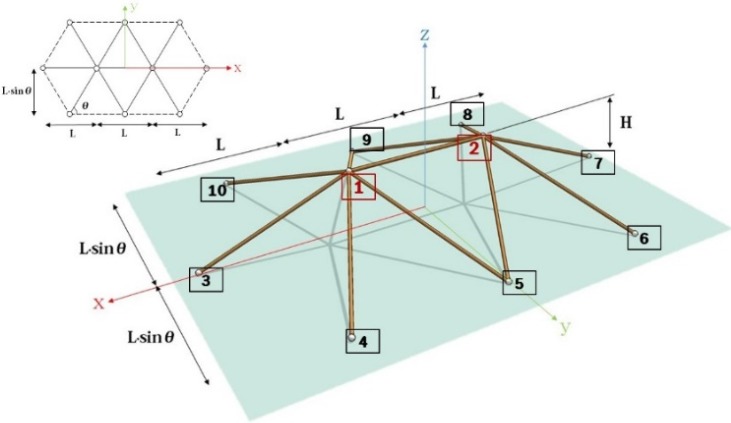
Shape of DFN model.

The second exemplar case structure is double-free-nodes (DFN) model as shown in Figure 10 [14,15]. The adopted model is composed of 10 nodes and 11 elements, and node 1 and 2 are free while the others are clamped. This model is more complex than the first one. In this study, the vertical displacements of node 1 and 2 (*i.e*., $D_{Z1}$ and $D_{Z2}$) are only considered to simplify the problem. In this case of DFN model, the governing equation, described by Equation (21), can be driven. In the governing equation, ${\text{ω}}_{0}=\sqrt{\left(5EAH^{2})/(m{\text{α}}^{3}L^{3}\right)}$ and $\alpha =L_{e}/L$. Where, $L_{e}$ is a length of an inclined element as shown in Figure 10.

(21) $$\ddot{D_{Z1}}+2{\text{ω}}_{0}h\dot{D_{Z1}}+{\text{ω}}_{0}^{2}D_{Z1}+\frac{1}{m}\left(k_{2}D_{Z1}^{2}+k_{3}D_{Z1}^{3}+k_{4}D_{Z1}D_{Z2}^{2}+k_{5}D_{Z1}^{2}D_{Z2}+k_{6}D_{Z2}^{3}-F\right)=0$$

The coefficients of Equation (21) are as follow:

(22) $$k_{1}=\frac{5EAH^{2}}{{\text{α}}^{3}L^{3}}\;,\;\;k_{2}=-\frac{7.5EAH}{{\text{α}}^{3}L^{3}}\;,\;\;k_{3}=\frac{0.5EA}{L^{3}}+\frac{2.5EA}{{\text{α}}^{3}L^{3}},\;k_{4}=\frac{1.5EA}{L^{3}}\;,\;\;k_{5}=-\frac{1.5EA}{L^{3}}\;,\;\;k_{6}=-\frac{0.5EA}{L^{3}}$$

where Young’s modulus (E), the density (ρ), the cross-sectional area (A) and span parameter (L) are equal to those of SFN model.

**Figure 11:**
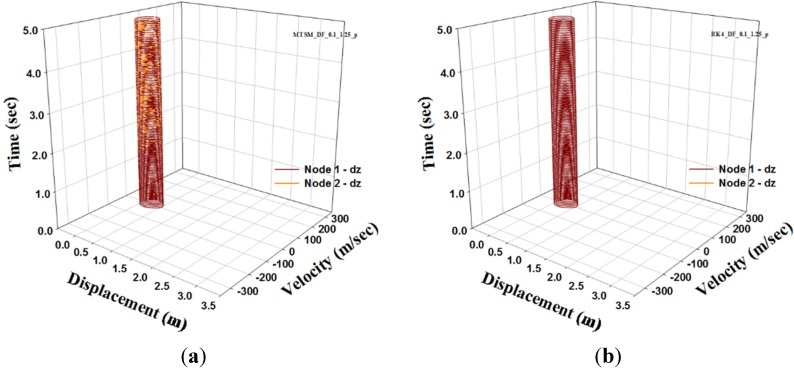
Phase diagram of DFN model. (Perfect case): (**a**) TSM; (**b**) RK4.

**Figure 12:**
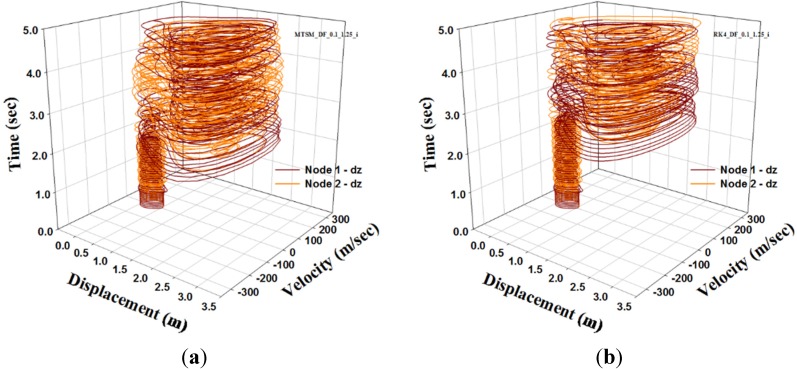
Phase diagram of DFN model. (Imperfect case): (**a**) TSM; (**b**) RK4.

In this section, the shape parameter μ = 0.1, *i.e.*, (H) = 1 m, and load level F=1300kN are considered. To analyze DFN model using TSM, let us consider the parameter n = 7 and the step $t^{\text{int}}=0.001$. For the comparison of the analysis results, the RK4 solution for $t^{\text{int}}=0.001$ is adopted. To observe a dynamic buckling phenomenon, we consider two different cases; one is a perfect shape and the other is an imperfect one. For the second case, 0.1% of *H* is applied to account for the initial imperfection. The analysis results using TSM and RK4 are as shown in Figure 11 and Figure 12.

In Figure 11 and Figure 12, we present a comparison between TSM and RK4 for both cases. In the figures, we observe that the attractors of TSM and RK4 agree with each other. For the first case, the result figure shows that a periodic orbit is observed, *i.e.* limit cycle. But Figure 12a in consideration of initial imperfection shows that a strange attractor is observed and displacement rapidly changes due to the influence of coupling under asymmetric imperfection. This figure indicates that the shallow DFN model is very sensitive to the initial condition. Generally, the DFN model is well known as a shallow space truss dome which is sensitive to the initial condition.

## 5. Conclusions

This paper described the nonlinear dynamic analysis of a steel space truss using TSM and investigated the dynamic instability under various excitations. The governing equations are formulated by considering geometrical nonlinearity, where an accurate analytical solution with a relatively long time-step could be obtained using the high-order TSM. In the investigation, the nonlinear dynamic response and trajectory of the phase space were analysed with step, sinusoidal and beating excitations. By analysing the response of the adopted models, the attractors and maximum displacement could delineate the characteristics of dynamic snapping, which occurs in a shallow shell under various excitations, and the change to an asymptotically stable state of the model with different levels of damping was well reflected. In investigating the dynamic instability of the SFN model, dynamic buckling occurred at approximately 77% of the static buckling with a step excitation. The dynamic buckling was approximately 83% of the static buckling when damping was considered, and the same result was obtained for all rise-span ratios (μ). The buckling load was lower with the model under periodic excitations compared with that of the model under a step excitation, and the model under a beating excitation reacted more sensitively.
